# Enhancing online learning engagement: teacher support, psychological needs satisfaction and interaction

**DOI:** 10.1186/s40359-025-03016-0

**Published:** 2025-07-01

**Authors:** Jinze He, Qiang Wang, Hanjin Lee

**Affiliations:** 1 Department of Digital Management, Graduate School, Korea University, Seoul, South Korea; 2 Handong University, Pohang, South Korea

**Keywords:** Online learning, Interaction, Self-determination theory, Learning engagement, Perceived teacher support, SEM

## Abstract

In the post-pandemic era, college students continue to embrace online learning due to its flexibility and accessibility. Grounded in self-determination theory, this study investigates how perceived teacher support influences students’ satisfaction of autonomy, competence, and relatedness, which in turn affects their interaction and engagement in online learning. Using data from 408 college students and analyzed through PLS-SEM, findings reveal that teacher support enhances intrinsic motivation, promoting interaction and learning engagement. Autonomy had the strongest influence on engagement. This research contributes to the integration of SDT with online learning frameworks and provides practical insights for educators and policymakers.

## Introduction

Universities have shown higher interest in undertaking educational activities online amid the COVID-19 pandemic [2, 7], and even in the post-pandemic era, the passion for using online learning keeps growing among college students and educational institutions. It is because both educational institutions and students have recognized the unique advantages of online learning, including flexibility [48], diverse learning styles [57], and self-pacing [19]. However, the shortcomings of lack of interaction [46] and resulting weak learning engagement also have been challenges for achieving better performance in online learning.

While previous studies have extensively explored learning engagement in online learning [8, 11, 17, 40], their typical focus is on the direct correlation between SDT learning engagement, while the antecedents and consequences associated with satisfying basic psychological needs are often overlooked. Additionally, few studies consider the role of interaction within the self-determination theory framework. To address this knowledge gap, this paper empirically examines factors that impact online learning interactions and engagement among college students.

## Literature review

### Learning in Self-determination Theory (SDT)

Self-determination theory (SDT) suggests that autonomy, relatedness, and competence are the three fundamental psychological needs of individuals [10]. They significantly contribute to enhancing human beings’ integration, constructive social development, and personal well-being [42]. In terms of autonomy, it means an individual’s psychological need for being a causal manager of one’s own life and to act in coordination with one’s integrated self. The relatedness originally indicates people's inborn willingness to be connected and significant to others. The concept of competence reflects people’s needs to control the result and enjoy a feeling of mastery [42]. When the three needs are satisfied, individuals enter a phase of optimal growth, well-being, and intrinsic motivation. Conversely, when they are thwarted, it can lead to weakened motivation and well-being [42].

Establishing a supportive environment can substantially boost individuals'motivation to commit, invest effort, and attain higher levels of performance, whether in educational or non-educational settings [42]. Research has shown that Self-Determination Theory (SDT) aids learners in transforming extrinsic motivation into intrinsic motivation, regulating their learning behavior from a state of amotivation [49]. Many studies have incorporated Self-Determination Theory into their research, based on this theoretical framework, confirming its critical significance in satisfying students'basic psychological needs and boosting learning motivation and outcomes in a variety of educational contexts [14, 16, 45, 49, 54].

### Prior study on online learning

#### Features of online learning

Online learning refers to a mode of education where instruction and content are delivered primarily over the internet, allowing students to learn without being physically present in a traditional classroom setting. It can take several forms, ranging from totally online programs that don't require in-person meetings to hybrid or blended programs that mix online learning with in-person instruction. [29]. Compared with traditional face-to-face learning modes, online learning has a set of advantages, including flexibility (students can get rid of the limitation of location and time) [48], diverse learning styles (online learning can cater to different learning styles with various tools and resources.) [57], and self-pacing (learn at one’s own speed or pattern) [19]. These features enable students to continue using online learning, even in the post-pandemic era when most courses have returned to face-to-face instruction.

Simultaneously, researchers have extensively examined the limitations of online learning, including factors such as a lack of interaction [46], technical challenges [1], the need for self-discipline [59], and limited practical experience [26]. These challenges have spurred interest in investigating methods to enhance students'interaction and motivation within the online learning environment [34].

#### Learning engagement in online learning

Learning engagement entails the consistent allocation of effort by students toward educational activities that directly contribute to expected academic achievements [9]. In the context of online learning, it encompasses the extent of students'attention, curiosity, interest, and passion during the learning process. [18]. It encompasses social, emotional, cognitive, and behavioral involvement in learning activities [12]. Engaged learners are more likely to be motivated, persist in their studies, and achieve better academic outcomes [4]. Paruthi and Kaur [35] defined four categories of online learning engagement: conscious attention, affection, enthused participation, and social connection, which provides a framework to assess the students'engagement in online learning.

Numerous studies have confirmed that satisfying students'basic psychological needs can have a positive impact on online learning engagement [8, 11, 17, 40]. When students'psychological needs for autonomy, relatedness, and competence are met, they become more intrinsically motivated to persist in the affective and cognitive aspects of online learning. However, many of these studies focus solely on the direct correlation between SDT and learning engagement, disregarding the examination of antecedents and consequences related to learners'satisfaction of basic psychological needs. Our study aims to address the knowledge gap by incorporating the antecedents and consequences of the three basic psychological needs into the research scope. We seek to explore the broader conceptual framework connecting SDT and online learning engagement.

#### Interaction in online learning

Interaction is a fundamental component of the learning process, regardless of the mode of instruction, especially in the online learning environment [24]. Research has revealed the dynamics within the virtual learning environment, including three types of interactions: student-to-student, student-to-teacher, and student-to-content [32], and these three types of interactions are confirmed to be positively related to learning engagement [47, 53]. In summary, when students are more motivated to interact, they are more engaged in the learning process. When students actively interact and engage in online learning, it positively contributes to their intention to use online learning, academic achievement, and overall satisfaction [15, 50, 53]. While interaction has traditionally been conceptualized as multidimensional, recent empirical studies suggest that the different types of online interaction are highly interrelated and contribute collectively to student engagement [28, 53]. Students often experience interaction holistically, rather than distinguishing between specific types, which further supports the validity of a single-factor model [30]. Therefore, in this study, interaction in online learning is conceptualized as a unidimensional construct to reflect its integrated role in fostering student engagement.

While previous studies have explored the partial relationship among interaction, learning engagement, and self-efficacy, there remains a gap in research that integrates these factors into a unified model, incorporating the three basic psychological needs.

#### Perceived teacher support in online learning

The definition of perceived teacher support is how supportive students recognize their teachers'stances and activities towards their academic performance and daily well-being [56]. This concept encompasses two dimensions: perceived teacher academic support and perceived teacher emotional support [23, 37]. Specifically, teacher academic support is defined by students'perceptions of their teacher's investment in their learning journey and their own knowledge acquisition. Conversely, teacher emotional support measures the degree to which students sense their teacher's sincere concern for their personal well-being [23].

The self-determination theory states that by meeting the three fundamental psychological needs of autonomy, relatedness, and competence, the external environment can increase intrinsic motivation, make it easier for external motivation to be internalized, and maintain active involvement [10]. It's also worth noting that the perceived level of teacher support can influence various student outcomes, from academic achievement to emotional well-being [36, 38, 42]. For instance, perceived teacher support has been found to be related to student engagement, with higher levels of support leading to increased academic engagement. [44]. An et al., [5] investigated student motivation to participate in online learning and found that it increases with teacher support. However, the study did not separately assess the specific contributions of the three fundamental psychological needs—autonomy, relatedness, and competence—possibly neglecting their individual impacts.

## Objectives and hypotheses

### Research objectives

**Fig. 1:**
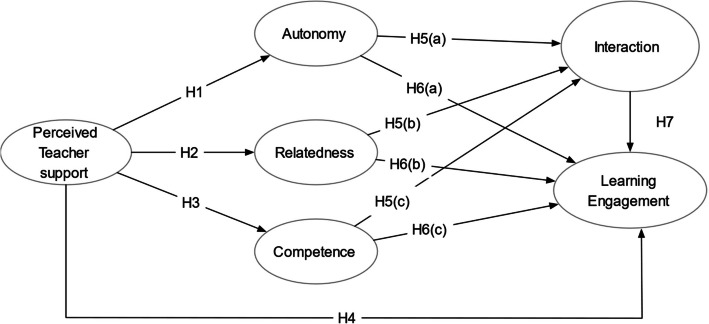
Conceptual model

This study addresses a knowledge gap by examining how the three basic psychological needs—autonomy, relatedness, and competence—affect responses to perceived teacher support and influence interaction and engagement in virtual learning. The goal is to create a conceptual framework to help educators and platform operators better understand online learners’ psychological processes, thereby improving course design and delivery. Specifically, the study investigates the relationships between perceived teacher support, these psychological needs, online interaction, and learning engagement, as illustrated in Fig. 1.

### Perceived teacher support and basic psychological needs

Perceived teacher support, recognized as a crucial external factor for students, has been shown to enhance students'emotional and academic well-being [37]. Previous studies have demonstrated that it satisfies students'needs for autonomy, relatedness, and competence. Additionally, perceived teacher support can serve as a source of emotional and behavioral motivation by enhancing students'self-efficacy [3, 5, 56, 58].

Based on this, we hypothesize:

- H1: Perceived teacher support positively influences students’ needs satisfaction for autonomy.
- H2: Perceived teacher support positively influences students’ needs satisfaction for relatedness.
- H3: Perceived teacher support positively influences students’ needs satisfaction for competence.

### Perceived teacher support and engagement

Studies have revealed that as students perceive teachers supporting them emotionally or academically, they are more motivated to invest more effort into online learning [5] since they feel cared about, supported, and encouraged during the process of online learning. Teacher support in online learning is primarily designed to improve learning outcomes, while learning engagement is a capital antecedent of learning achievement [4], so we propose.

- H4: Perceived teacher support is positively related to online learning engagement.

### Effect of three basic psychological needs on interaction

As previously discussed, interaction is a crucial element of online learning. Students are more likely to participate in interactive activities, such as engaging in online group discussions, answering questions from classmates or teachers, and sharing their study progress when they are intrinsically motivated [18]. So based on this knowledge, we hypothesize:

- H5(a): Students’ needs for autonomy are positively related to interaction.
- H5(b): Students’ needs for relatedness are positively related to interaction.
- H5(c): Students’ needs for competence are positively related to interaction.

### Basic psychological needs and online learning engagement

Studies have thoroughly investigated the causal relationship between the three psychological needs and their effect on learning engagement. When students feel autonomous, related, and competent, they become intrinsically motivated and are willing to actively persist and commit to the online learning process [42]. So, we make the following hypotheses:

- H6(a): Students’ needs for autonomy are positively related to learning engagement.
- (b): Students’ needs for relatedness are positively related to learning engagement.
  H6(c): Students’ needs for competence are positively related to learning engagement.

### Online interaction and learning engagement

Studies have confirmed that online learning interaction positively influences online learning engagement [27, 47]. Interactions enhance students'sense of involvement, immersion, and activeness, thereby fostering high-quality online learning engagement [18]. Thus, we advocate.

- H7: Online learning interaction is positively related to online learning engagement.

## Research methods

### Scales development

To collect data from college students participating in online learning, we employed a questionnaire containing 31 items on a 5-point Likert scale (see Table 2). These items assess the six variables in the present study, along with some demographic questions. All items were adapted from previous studies and revised to align with the context of online learning. For perceived teacher support, eight items were adapted from two papers [37],A. M. [41]. Fifteen items were adapted from two studies(R. M. [43],Van Den [51]), with five items allocated to each of the autonomy, relatedness, and competence dimensions. Nine items adapted from three papers [28, 53, 55], were used for measuring online interaction. Four items adapted from prior studies were used for measuring online learning engagement [12] as shown in Table 2. To ensure reliability and content validity, the questionnaire was developed by adapting established measurement scales and subjected to rigorous validation procedures. Initially, it was translated from English into Chinese and then back-translated to confirm conceptual equivalence. The final Chinese version was reviewed and approved by two PhD candidates with expertise in the relevant fields.

### Data collection

An online questionnaire survey was conducted in April 2025 using Wenjuanxing, a widely used national survey platform operated by a Chinese service provider. The survey was distributed using a non-probability snowball sampling method [13]. The researchers initially contacted familiar teachers via email, asking them to help circulate the survey among college students with experience in online learning. A total of 500 questionnaires were distributed. After excluding invalid responses—specifically, those completed in under two minutes or answered indiscriminately—we obtained 408 valid responses, yielding a response rate of 81.6%.

To protect participant privacy and data security, several precautions were taken. Prior to starting the survey, respondents were provided with a brief explanation of the research objectives and an outline of the questions. Participation was entirely voluntary, and all responses were anonymized to maintain confidentiality. No personally identifiable information was collected, and all data were used solely for academic purposes.

## Results

The demographic statistics shown in Table 1, of the *N* = 408, 220 (53.92%) are males, and 188 (46.08%) are females. Most respondents were aged from 18 to 25 years old (*N* = 379, 92.89%). The grades of these college students are 77 freshmen (18.87%), 123 sophomores (30.15%), 114 juniors (27.94%), and 94 seniors (23.04%), respectively.

**Table 1:** Demographic statistics (*N* = 356)

Variables	Frequency	Percent (%)
Gender		
Male	220	53.92
Female	188	46.08
Age group		
18–25	379	92.89
26–30	29	7.11
Grade		
Freshmen	77	18.87
Sophomore	123	30.15
Junior	114	27.94
Senior	94	23.04
City Tiers		
First tier	15	3.68
New first tier	175	42.89
Second tier	31	7.60
Third tier	27	6.62
Other	160	39.21

We applied the Partial Least Squares (PLS) method using Smart-PLS 3 software. PLS has been proven to be efficient in handling small sample sizes and complex conceptual models [20]. Given that this study involves six constructs with relatively intricate connections, we use PLS for this empirical research.

### Common method bias

Prior to evaluating the structural equation model (SEM), we employed SPSS 27 for Harman's one-factor test to identify any common method bias (CMB). The results indicated that a single factor accounted for 33.26% of the total variance, suggesting that common method bias does not pose a concern [39].

### Measurement model

As shown in Table 2, all constructs have Cronbach’s alpha and composite reliability that meet the thresholds(> 0.7) [52]. We then examined the convergent and discriminant validity by conducting the four tests recommended by Voorhees et al., [52]. Test 1: As shown in Table 3, AVEs of all constructs exceeded 0.5, surpassing the threshold value suggested by Voorhees et al., [52]. Test 2: As shown in Table 3, the square root of the AVE values for each construct (highlighted in bold) exceeded their respective off-diagonal correlations, as suggested by Voorhees et al. [52]. Test 3: The loading for each item was above 0.70, in line with the findings of Voorhees et al. [52]. Test 4: Based on the methodology of Henseler et al., [21], we evaluated the Heterotrait-Monotrait ratio (HTMT) of correlations. This was done by dividing the average correlations between different constructs by the average correlations of indicators within the same construct. All HTMT values were below the 0.85 threshold, as recommended by Voorhees et al. [52]. Collectively, these findings suggest satisfactory convergent and discriminant validity of the measurements.

**Table 2:** Construct measurement and confirmatory factor analysis

			Mean	SD	Factor Loadings	Cronbach’s α	CR	AVE
Perceived teacher support	PTS1	My teacher(s) truly get how I feel about the situation	3.284	1.233	0.773	0.910	0.927	0.614
	PTS2	My teacher(s) try to support me when I'm depressed or anxious	3.223	1.286	0.780			
	PTS3	I can count on my teacher(s) for help when I need it	3.272	1.350	0.792			
	PTS4	My teacher(s) value my opinion	3.353	1.322	0.802			
	PTS5	My teacher(s) care about how much I learn	3.350	1.326	0.798			
	PTS6	My teacher(s) enjoy seeing my work	3.228	1.392	0.795			
	PTS7	My teacher(s) want me to do my best in learning	3.311	1.249	0.746			
	PTS8	My teacher(s) like to help me learn	3.225	1.327	0.782			
Autonomy	ANS1	I have the freedom to choose how I want to approach the assignments for my online course	3.319	1.296	0.815	0.866	0.903	0.650
	ANS2	I am free to choose how I want to complete my online learning assignment	3.301	1.284	0.802			
	ANS3	I have many options to choose how I want to approach my online education	3.382	1.310	0.804			
	ANS4	I believe I have a lot of control over how my online education is carried out	3.348	1.271	0.800			
	ANS5	I have the freedom to voice my thoughts and opinions on what I'm learning online	3.373	1.430	0.810			
Relatedness	RNS1	I feel like I'm connected to other individuals through online learning	3.292	1.319	0.797	0.878	0.911	0.673
	RNS2	It feels like a group when I learn online	3.409	1.242	0.817			
	RNS3	When I'm online with my classmates, I don't feel alone	3.380	1.417	0.832			
	RNS4	During online learning, I can discuss topics that are truly important to me with other individuals	3.434	1.383	0.836			
	RNS5	I get along well with other individuals at online learning	3.446	1.340	0.819			
Competence	CNS1	I master my tasks at online learning	3.350	1.237	0.817	0.864	0.902	0.648
	CNS2	I have the ability to complete my assignments for online learning	3.343	1.255	0.801			
	CNS3	I have faith in my ability to do the assignments for my online course	3.333	1.296	0.828			
	CNS4	My completion rate for online learning assignments is high	3.400	1.377	0.805			
	CNS5	I feel like I can complete even the trickiest online learning assignments	3.326	1.323	0.774			
Interaction	ITR1	I interact with instructors on the issues of learning on the online learning platform	3.311	1.607	0.820	0.948	0.956	0.706
	ITR2	I inquire of instructors about learning advice on the online learning platform	3.314	1.524	0.788			
	ITR3	I discuss learning tasks with instructors on the online learning platform	3.353	1.537	0.826			
	ITR4	I interact with the online learning platform on my learning activity	3.316	1.447	0.828			
	ITR5	I organize my learning course on the online learning platform	3.348	1.560	0.853			
	ITR6	I release my learning content on the online learning platform	3.306	1.605	0.857			
	ITR7	I share learning information with other students on the online learning platform	3.289	1.524	0.865			
	ITR8	I exchange knowledge with other students on the online learning platform	3.338	1.582	0.851			
	ITR9	I interact with other students about the learning issues on the online learning platform	3.301	1.515	0.826			
Learning engagement	LE1	After the online lesson, I study the relevant learning material on my own	3.287	1.430	0.830	0.861	0.906	0.706
	LE2	I often engage with other learners by responding to questions, participating in discussions, and sharing materials	3.346	1.417	0.847			
	LE3	When I take an online course, I plan a learning schedule	3.390	1.409	0.842			
	LE4	Engaging in online learning makes me feel happy	3.267	1.460	0.841			

*SD* Standard deviation, *α* Cronbach’s alpha, *CR* Composite reliability, *AVE* Average variance extracted

**Table 3:** Analysis of discriminant validity

	Autonomy	Competence	Interaction	Learning Engagement	Perceived Teacher Support	Relatedness
Autonomy	**0.806**					
Competence	0.379	**0.805**				
Interaction	0.356	0.427	**0.840**			
Learning Engagement	0.466	0.387	0.419	**0.840**		
Perceived Teacher Support	0.405	0.335	0.407	0.443	**0.784**	
Relatedness	0.385	0.395	0.445	0.387	0.359	**0.820**

The AVE's square root is indicated by the bolded number

### Hypothesis testing

We evaluated the structural model with the bootstrapping method for hypothesis testing. Initially, we used Smart-PLS 3 to calculate the Variance Inflation Factor (VIF). No VIF values exceeded 3, indicating the absence of collinearity problems [20]. The coefficient of determination (R^2^) of interaction and learning engagement is 29.0% and 35.4%, respectively, indicating an acceptable explanatory power for the model [20].

**Fig. 2:**
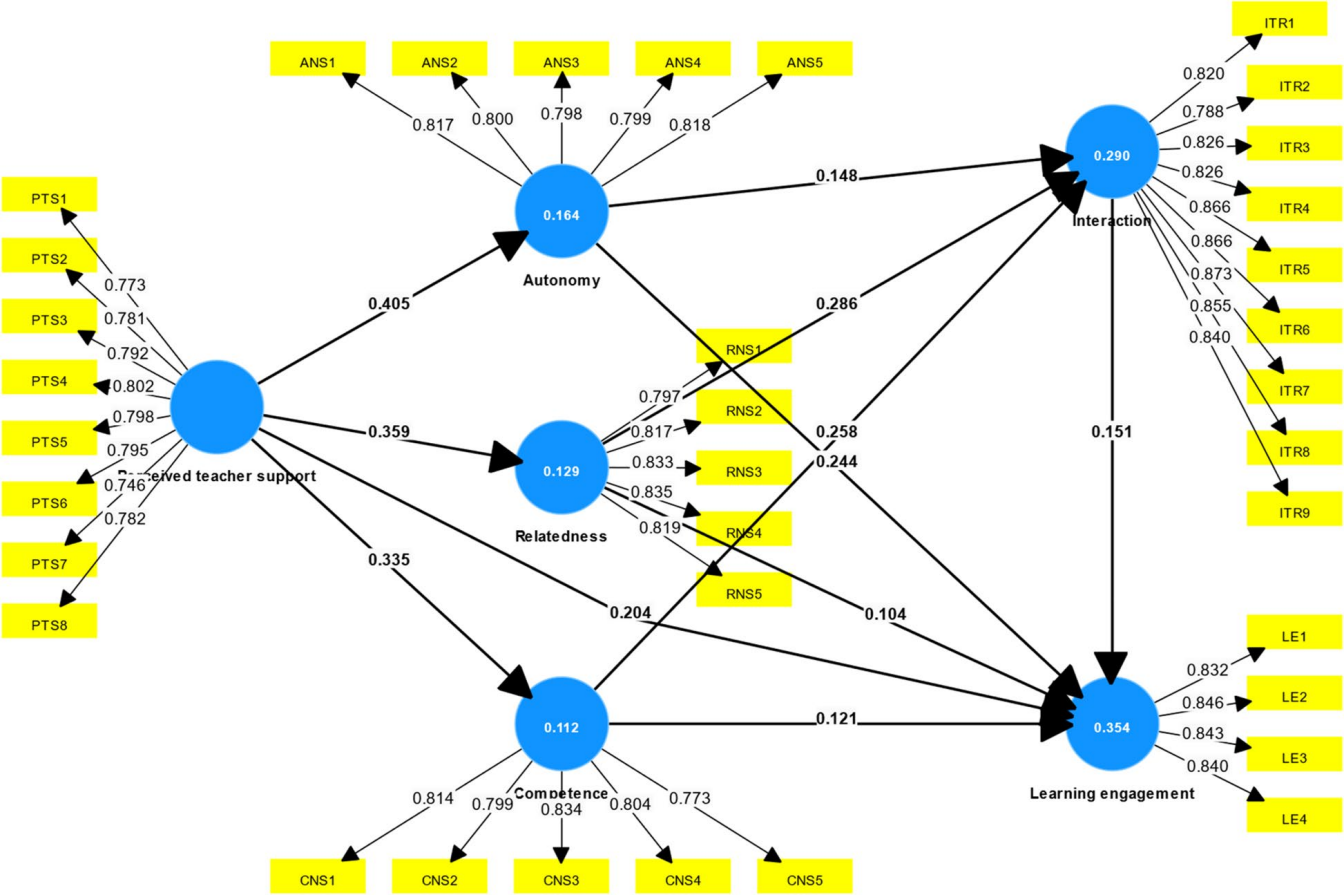
Path coefficient and R^2^

Based on the findings of the structural model, all hypotheses were accepted, as shown in Table 4, with all *p*-values significant at the 0.05 level, with all path coefficients shown as Fig. 2. The path analysis results validate all the hypothesized relationships in our model. Perceived teacher support significantly enhances student autonomy (β = 0.405, *p* < 0.001), relatedness (β = 0.359, *p* < 0.001), and competence (β = 0.335, *p* < 0.001), and it also has a direct positive effect on learning engagement (β = 0.204, *p* < 0.001). In addition, the psychological needs of autonomy, relatedness, and competence significantly drive online interaction with coefficients of 0.148 (*p* = 0.002), 0.286 (*p* < 0.001), and 0.258 (*p* < 0.001), respectively. These needs further contribute directly to learning engagement: autonomy (β = 0.244, *p* < 0.001), relatedness (β = 0.104, *p* = 0.033), and competence (β = 0.121, *p* = 0.011). Finally, increased interaction itself positively influences learning engagement (β = 0.151, *p* = 0.004). Collectively, these findings suggest that teacher support boosts psychological need satisfaction, which in turn promotes student interaction and ultimately leads to higher engagement in online learning environments.

**Table 4:** Hypothesis testing

	Path Coefficient	Standard Deviation	T-values	*P*-Values	Results
Perceived Teacher Support—> Autonomy (H1)	0.405	0.042	9.654	0	Accepted
Perceived Teacher Support—> Relatedness (H2)	0.359	0.041	8.828	0	Accepted
Perceived Teacher Support—> Competence (H3)	0.335	0.042	7.921	0	Accepted
Perceived Teacher Support—> Learning Engagement (H4)	0.204	0.047	4.379	0	Accepted
Autonomy—> Interaction (H5(a))	0.148	0.049	3.042	0.002	Accepted
Relatedness—> Interaction (H5(b))	0.286	0.047	6.064	0	Accepted
Competence—> Interaction (H5(c))	0.258	0.051	5.101	0	Accepted
Autonomy—> Learning Engagement (H6(a))	0.244	0.048	5.055	0	Accepted
Relatedness—> Learning Engagement (H6(b))	0.104	0.049	2.129	0.033	Accepted
Competence—> Learning Engagement (H6(c))	0.121	0.047	2.551	0.011	Accepted
Interaction—> Learning Engagement (H7)	0.151	0.053	2.847	0.004	Accepted

## Discussion

This study applies self-determination theory to examine how college students’ perceptions of teacher support influence their psychological needs—autonomy, relatedness, and competence—and subsequently impact their interactions and engagement in online learning. Data analysis reveals that perceived teacher support, such as care, encouragement, and guidance, fosters greater satisfaction of these needs. Specifically, supportive teachers help students feel more autonomous, connected to peers, and competent in learning activities. This finding aligns with existing research showing that teacher support positively affects students'self-efficacy and learning motivation [3, 5, 56, 58]. Notably, our study goes beyond these findings, which provide further evidence that teacher support can intrinsically motivate students from the perspective of fulfilling their psychological needs.

The study shows that when students’ psychological needs for autonomy, relatedness, and competence are met, they become more intrinsically motivated and actively engaged in online learning. This motivation drives a natural desire to connect and interact with peers and teachers, as seen in their participation in group activities, expression of ideas, and collaborative efforts. Consequently, students invest more effort and focused attention in their learning, leading to better interaction and deeper engagement in the online learning environment. Besides, as the students are willing to interact with their course content, peers, and instructors, it further enhances students’ engagement with online learning, which is aligned with the results of previous studies [30, 53]. For example, when teachers organize peer review activities where students give and receive feedback, it enhances competence, relatedness, and autonomy simultaneously, and then enhances interaction and learning engagement consequently.

This finding extends the research conducted by Fang et al., [17] and Wang et al., [53]. Fang et al., [17] noted that the satisfaction of the basic psychological needs can enhance learning engagement in online settings, while Wang et al., [53] highlighted the effect of online learning interaction in increasing the level of online learning engagement. Our study expands on these findings by showing how the fulfillment of fundamental psychological needs and interactions in online learning work together to improve students'engagement in online learning.

## Implications

### Theoretical implications

This study expands the scope of online learning literature by examining psychological and behavioral aspects beyond the conventional focus on self-efficacy and learning motivation. Previous research has not extensively explored the mechanisms of the three basic psychological needs as drawn upon by the self-determination theory in response to perceived teacher support and their impact on interactions and learning engagement. Our study pioneers a framework for measuring the factors that influence students'online learning engagement from the perspective of the self-determination theory and online learning interaction.

Interestingly, the three basic psychological needs exert differential influences on interaction and learning engagement. Specifically, the need for relatedness appears to have a stronger effect on interaction than autonomy or competence, whereas the need for autonomy demonstrates a more substantial impact on learning engagement. These findings suggest that fostering a sense of belonging within study groups or classes can effectively enhance students’ willingness to interact with course content and companions. Conversely, stimulating students’ intrinsic motivation to learn—by supporting their autonomy—emerges as the most effective strategy for promoting engagement in online learning environments. Previous studies [17, 22] have also noted this phenomenon, highlighting how these psychological needs can differentially influence behavioral, emotional, and cognitive engagement.

### Managerial implications

Our study's findings suggest several managerial implications. Firstly, teachers'support should be given more emphasis as a crucial component of online learning. Given the inherent limitation of online learning in lacking face-to-face interaction [46], it becomes essential for teachers to actively engage in the student's learning process. They should closely monitor students'progress, identify challenges they may encounter, and provide prompt feedback tailored to their needs. This proactive approach can help students develop intrinsic motivation to actively participate in online learning interactions and foster greater engagement.

Secondly, interactions should be a priority during the course design and operation stages due to the fulfillment of the three psychological demands and as a precondition for online learning engagement. More resources should be provided by instructors and course administrators to improve interactions in online learning. This can be accomplished by taking steps like creating badge profiles or gamifying courses, both of which have been shown to greatly improve the online learning experience [6]. Moreover, research has shown that utilizing generative AI tools to develop online learning environments is an efficient method to enhance student interaction and engagement with the learning process. This is an emerging trend in education(H.J [25]). The significance of this trend becomes particularly evident when examined through the lens of the pre-, during-, and post-COVID-19 era. Prior to the pandemic, online learning was often viewed as a supplementary mode of instruction. However, during the COVID-19 crisis, educational institutions worldwide were forced to shift rapidly to fully remote learning, exposing both the limitations and potential of digital education. This unprecedented transition underscored the urgent need for innovative solutions to maintain learner engagement and instructional quality. In the post-COVID-19 era, as hybrid and fully online education continue to evolve, generative AI technologies offer promising tools to personalize learning, simulate real-life scenarios, and facilitate meaningful student interactions. Thus, the educators should also consider combining generative AI technologies into the design of online learning to meet the growing demand for flexible, engaging, and effective educational experiences.

## Conclusion, limitation, and further research

Drawing on self-determination theory, this research investigates the impacts of online learning interaction and engagement. It aims to fill the knowledge gap left by prior studies and to enhance the theoretical model. This study integrates the three fundamental psychological needs—autonomy, relatedness, and competence—with aspects of online interaction. In conclusion, we have confirmed the positive effects and interaction mechanisms of these variables. Our findings suggest that perceived teacher support significantly enhances students'satisfaction with autonomy, relatedness, and competence, consequently leading to higher-quality online learning interaction and increased learning engagement.

This study has several limitations. First, the sample is solely pooled from college students in China, limiting its generalizability to other populations with diverse occupations and cultures, potentially resulting in different online learning characteristics. Second, the study lacks a longitudinal design to measure changes in students'online learning interaction and engagement before and after experiencing varying levels of perceived teacher support and satisfaction of basic psychological needs. At last, this study uses a unidimensional construct for online interaction, which may overlook the unique roles of learner-learner, learner-instructor, and learner-content interactions.

Future research could explore these interaction dimensions separately for more nuanced insights into their effects on student engagement and may also consider cross-sectional or longitudinal studies to examine how online interaction and engagement evolve across different learner groups and stages of education.

## Data Availability

The datasets generated and/or analyzed during the current study are available from the corresponding author upon reasonable request.
